# Supplementary material for: On-Axis Optical Trapping with Vortex Beams: The Role of the Multipolar Decomposition
Source: ACS Photonics. 2024 Jan 31;11(2):626–33. doi: 10.1021/acsphotonics.3c01499 (PMC10885202; doi:10.1021/acsphotonics.3c01499)
Supplement: Supplementary file 1 — ph3c01499_si_001.pdf [file ph3c01499_si_001.pdf]

Supporting Information for On-axis optical trapping with vortex beams: the role of the multipolar decomposition

Iker Gómez-Viloria,[*,1] Álvaro Nodar,[1] Martín Molezuelas-Ferreras,[1] Jorge Olmos-Trigo,[1] Ángel Cifuentes,[1] Miriam Martínez,[1] Miguel Varga,[1,2] and Gabriel Molina-Terriza[*,1,2,3]

[1]*Centro de Física de Materiales (CFM), CSIC-UPV/EHU, Paseo Manuel de Lardizabal 5, 20018 Donostia-San Sebastián, Spain*

[2]*Donostia International Physics Center, Paseo Manuel de Lardizabal 4, 20018 Donostia-San Sebastián, Spain*

[3]*IKERBASQUE, Basque Foundation for Science, Maria Diaz de Haro 3, 48013 Bilbao, Spain*

* E-mail: iker_gomez@hotmail.com; gabriel.molina.terriza@gmail.com

Contents

Supporting Note 1. Theoretical description of electromagnetic fields and forces **3**

S1.1 Multipolar decomposition of electromagnetic fields 3

S1.2 Multipolar expansion of cylindrically symmetric optical systems 4

S1.3 Theoretical calculation of optical forces 5

Supporting Note 2. Description of the experimental setup **7**

Supporting Note 3. Trap stiffness measurements through Power Spectrum Density (PSD) method **11**

References **13**

Supporting Note 1. Theoretical description of electromagnetic fields and forces

S1.1 Multipolar decomposition of electromagnetic fields

As a first step, we consider an arbitrary monochromatic electromagnetic field with well-defined helicity. The incident electric field is written in terms of the well-defined helicity multipoles[1–3] as

$$\mathbf{E}_i^p = \sum_{j=1}^{\infty} \sum_{m_z=-j}^{j} D_j C_{j,m_z,p} \, \mathbf{A}_{j,m_z}^p \tag{1}$$

where j is the total angular momentum, m_z is the angular momentum in z direction and p is the helicity of the incoming light beam (for a non-focused light beam helicity is equal to the polarization). We also have that $D_j = i^j(2j+1)^{\frac{1}{2}}$ and $C_{j,m_z,p}$ are the Beam Shape Coefficients (BSC). The BSCs contain the transversal mode amplitude and the focusing information following the Aplanatic Lens Model,[4] and are defined as in reference[5] for a focus placed in the center of coordinates and in references[6,7] for the displaced case. $\mathbf{A}_{j,m_z}^p$ are the well-defined helicity multipoles, formed combining the electric and magnetic multipoles[1–3] as

$$\mathbf{A}_{j,m_z}^p = \frac{\mathbf{A}_{j,m_z}^{(m)} + ip\mathbf{A}_{j,m_z}^{(e)}}{\sqrt{2}}, \tag{2}$$

which are eigenstates of the helicity operator $\Lambda = \frac{\nabla\times}{k}$, respecting the relation $\Lambda\mathbf{A}_{j,m_z}^p = p\mathbf{A}_{j,m_z}^p$.

The scattered electric field has the contribution of both positive and negative helicities, and are expressed as

$$\mathbf{E}_{sc}^p = \sum_{j=1}^{\infty} \sum_{m_z=-j}^{j} D_j C_{j,m_z,p} \, (\alpha_j \mathbf{A}_{j,m_z}^p + \beta_j \mathbf{A}_{j,m_z}^{-p}), \tag{3}$$

where

$$\alpha_j = -\frac{a_j + b_j}{2} \quad \text{and} \quad \beta_j = \frac{a_j - b_j}{2}. \tag{4}$$

Here we have that α_j and β_j would play the role of the scattering coefficients of this basis, being a combination of the well-known Mie coefficients a_j and b_j.[8–11]

The magnetic field can be derived from the electric field using the relation $\mathbf{H} = -i\sqrt{\frac{\epsilon}{\mu}}\Lambda\mathbf{E}$ of Maxwell equations.

Finally, for our calculation of the forces the total electric and magnetic fields must be taken, which are derived as

$$\mathbf{E}_{tot} = \mathbf{E}_{in} + \mathbf{E}_{sc} \quad \text{and} \quad \mathbf{H}_{tot} = \mathbf{H}_{in} + \mathbf{H}_{sc}. \tag{5}$$

S1.2 Multipolar expansion of cylindrically symmetric optical systems

In this subsection, we are going to analyze the consequences of the on-axis configuration in a optical system with cylindrical symmetry. This is also the case when a spherical particle is optically trapped centred along the longitudinal axis (z) of a focused Gaussian or Laguerre-Gaussian beam, which is the main system under study in this work. We refer to this kind of trapping as "on-axis" configuration and it has deep implications for the multipolar decomposition of the electromagnetic field described in this work. The cylindrical symmetry implies that the incident E_i and scattered E_{sc} fields are eigenstates of the operator of rotations around z axis $R_z(\theta) = exp(-iJ_z\theta)$, what, at the same time, means that the m_z is well-defined in E_i and E_{sc}, leading to the eigenstate relations $J_zE_i = m_zE_i$ and $J_zE_{sc} = m_zE_{sc}$. In consequence, we have that each individual multipole forming these fields has the same fixed value of $m_z^* = L + p$, allowing to simplify their expressions as

$$\mathbf{E}_i^{p,on} = \sum_{j=|m_z^*|}^{\infty} D_j C_{j,m_z^*,p}^{on} \, \mathbf{A}_{j,m_z^*}^{p} \tag{6}$$

and

$$\mathbf{E}_{sc}^{p,on} = \sum_{j=|m_z^*|}^{\infty} D_j C_{j,m_z^*,p}^{on} \left(\alpha_j \mathbf{A}_{j,m_z^*}^{p} + \beta_j \mathbf{A}_{j,m_z^*}^{-p}\right). \tag{7}$$

Note that the notations of both electric fields and the BSCs include an "on" superscript, meaning that they are describing the on-axis configuration. The lower limit of the j summation has been also set to $|m_z^*|$ due to the properties of the spherical harmonics basis. This last effect suppresses a certain number of the lowest multipolar orders depending on the values of L (topological charge) and p (helicity) of the trapping beam, simplifying the multipolar analysis of this kind of systems.

On the other hand, the cylindrical symmetry along z would be broken if any relative displacement between the Gaussian or Laguerre-Gaussian beam and the spherical scatterer is performed along the transversal directions (x or y). This would require again the general description of the electric field with the complete summation of multipoles, described in Subsection S1.1.

S1.3 Theoretical calculation of optical forces

We employed the Maxwell Stress Tensor (T_{ij})[12] to calculate the optical force acting on a spherical scatterer. In particular, we have integrated T_{ij} over a sphere in the far-field surrounding the illuminated spherical particle. The i-component of the forces is given by

$$F_i = \oint T_{ij} n_j dA, \tag{8}$$

where

$$T_{ij} = \frac{1}{2}\,\mathbb{R}\left[\epsilon E_i E_j^* + \mu H_i H_j^* - \frac{1}{2}\epsilon(\mathbf{E}\cdot\mathbf{E}^* + Z^2 \mathbf{H}\cdot\mathbf{H}^*)\,\delta_{ij}\right]. \tag{9}$$

Here $\mathbf{E}$ and $\mathbf{H}$ denote the total electric and magnetic field, n_j is a unitary vector that is normal to the differential of the area of a sphere surrounding the sample dA. Since in far-field the electromagnetic fields only contain transverse components, the first two terms of

the left-hand side of Eq. (9) do not contribute to the optical force, yielding

$$\mathbf{F} = -\frac{r^2}{4}\,\mathbb{R}\int \epsilon(\mathbf{E}\cdot\mathbf{E}^* + Z^2\mathbf{H}\cdot\mathbf{H}^*)\,\hat{\mathbf{r}}\,d\Omega. \tag{10}$$

where Ω denotes the solid angle. Finally, we analytically calculated these integrals as in [13] to obtain the optical forces under the illumination of LGs with well-defined helicity and total angular momentum.

Supporting Note 2. Description of the experimental setup

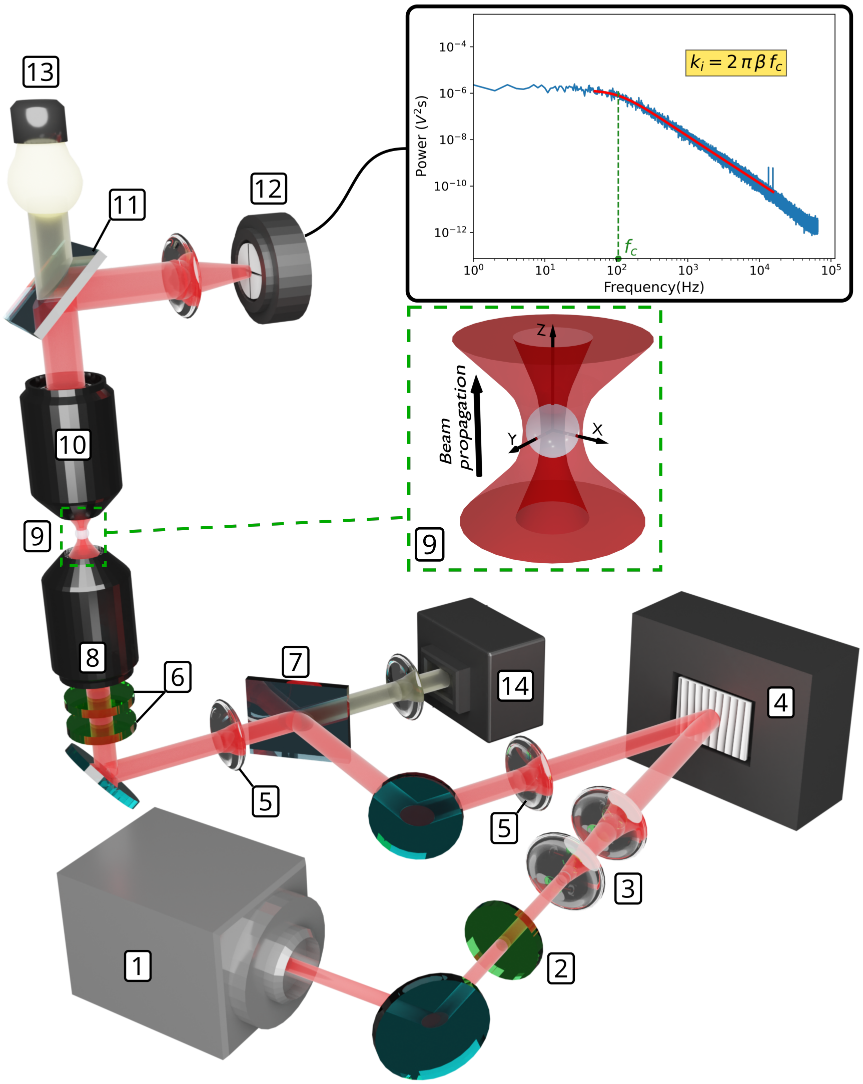

Supporting Figure 1: **Experimental optical tweezers setup.** The main subsystems contained in the setup are the wavefront phase modulation system, the imaging system of the X/Y plane of the trapping region and the trap stiffness constant ($\kappa_{x/y}$) measurement system.

At the beginning of the optical system, we have the laser source (element 1 of Supporting Fig. 1), an erbium-doped fiber diode. It emits a CW TEM00 of, approximately, 1 mm of diameter, at a wavelength of 976 nm. Note that it emits a variable optical power depending on the current applied to the laser diode.

Next, to create light vortex beams, we modulate the laser source's wavefront via a high-

resolution LCOS-SLM (element 4) operating in an off-axis configuration. This device modulates the phase of the wavefront of the incoming light pixel by pixel, guided by a set of phase shift masks. The fundamental pattern for generating vortex beams superposes an azimuthal phase onto a blazed grating, so that the vortex beam forms in the first diffraction order, and is easily separated from stray light. Elements 2 and 3 are mainly devoted to adapting the laser beam to the requirements of the LCOS-SLM. First, its polarization is set with a half-waveplate (element 2). Then, the beam size is adjusted with a beam expander (element 3) formed by two plane-convex achromatic doublets with focal distances 25.4mm and 75mm. Note that the beam expander includes a spatial filter using a pinhole of $30\mu m$ of diameter to eliminate higher frequencies of the beam.

After the phase modulation is applied, the beam wavefront at the SLM's screen plane is reconstructed onto the back focal plane of the focusing objective (element 8) using a 4f system (element 5). This ensures that the back aperture of the objective is filled with a constant amount of light when the different beam modes are employed and allows us to achieve a tight diffraction limited vortex beam with little to no distortion. For the trapping experiments shown in this work the back aperture with a diameter $D \simeq 6mm$ was filled with Gaussian-shaped beam of $1/e^2$ halfwidth $w_0 \simeq 2mm$. The value of w_0 was chosen to be smaller than the radius of the back aperture of the microscope objective to focus as much beam power as possible,[14] but specially to reduce the interference between the beam and the edges of microscope entrance. Note that, along the 4f line, between the SLM and the focusing elements, the polarization of the beam is adjusted to pure circular using a quarter-wave plate and an additional half-wave plate (elements 6). Ideally, a single quarter-wave plate applied to a linearly polarized beam would be able to generate perfect circular polarization, but experimentally, only the combination of both elements provides the fine-tuning of the polarization state required for this work. Along the 4f system, we can also find a dichroic mirror (element 7) that reflects the 976 nm trapping laser towards the objective lens (element 8) and transmits the visible illumination light going to the camera (element

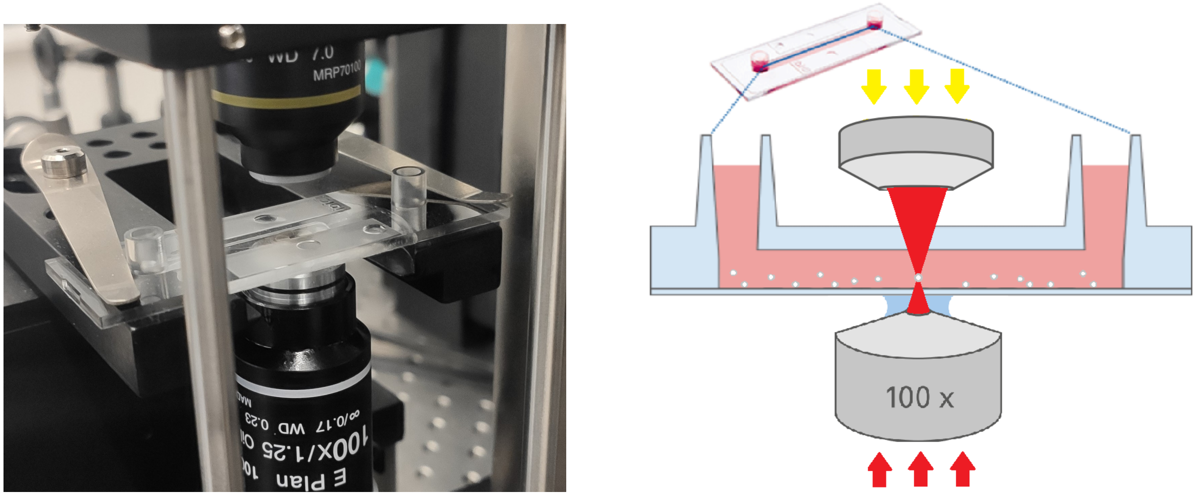

(a) (b)

Supporting Figure 2: **Experimental trapping environment.** a) Picture of the experimental trapping elements. It is possible to differentiate the micrometric precision positioner holding the micro-channel slide, the trapping laser focusing objective lens (bottom) and the condenser objective lens (top). b) Sketch of the experimental trapping elements. In this drawing it is possible to see the configuration of the micro-channel slide and the way it contains the monodisperse suspension of spherical silica particles in water.

14).

The next part of our setup is formed by the optical trap (element 9) and the elements conforming to it. Here, the laser beam is tightly focused by the previously mentioned 100X oil immersed objective lens of NA=1.25 and WD=0023mm (element 8), which is capable of producing a beam waist with sizes $< 1\mu m$.

In our case, the trapped samples are silica (SiO_2) spherical particles with a diameter of $2\mu m$. These spherical particles are part of a monodisperse suspension in water, which is confined in a micro-channel slide of 0.4mm of height made of a transparent polymer (see Supporting Fig. 2). Due to gravitational forces, these silica beads fall right to the bottom of the channel. Hence, this plane must be finely aligned with the focal plane of our objective lens to make the particles interact with the highest intensity part of our laser beam to get trapped. For this purpose, we adjust the position of the slide with a micrometric precision positioner, aided, at the same time, by the imaging system that will be explained shortly. Supporting Fig. 3 shows two pictures obtained through the imaging system of the trapping

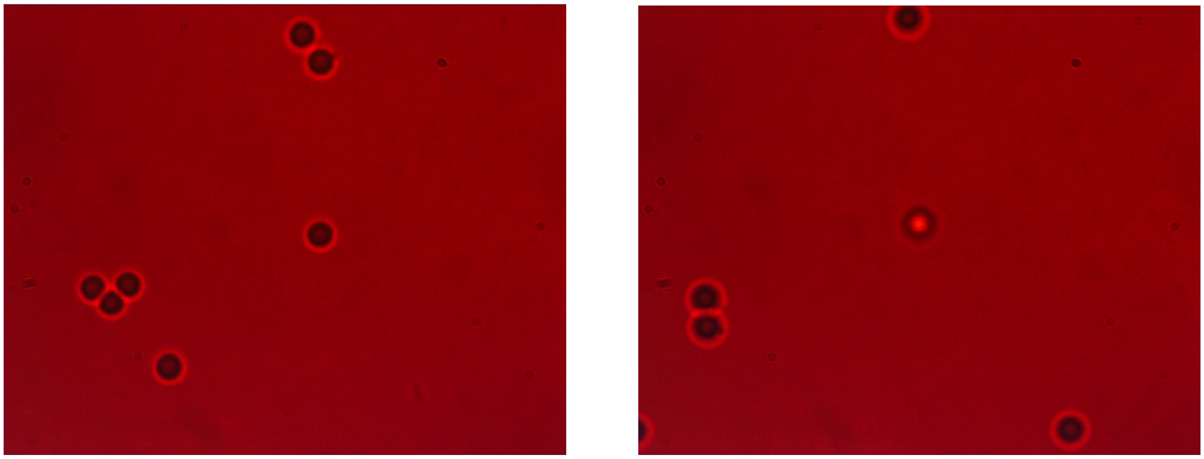

(a) (b)

Supporting Figure 3: **Imaging of the X/Y trapping plane** a) SiO_2 particles in the bottom of the micro-channel slide in the absence of trapping light. b) SiO_2 particles in the bottom of the micro-channel slide, but in this case one of them (the one in the centre) is optically trapped. Note that the trapped particle is slightly out of focus with respect to the imaging plane.

plane of the experimental setup. Note that the monodisperse suspension of silica particles in water must be carefully diluted in order to reach the proper concentration of particles in the bottom of the micro-channel slide. If the number of particles is too high in this plane, trap stiffness measurements could easily be spoiled by the interference of other attracted particles.

After the laser light interacts with the trapped particle, the forward scattering is collected and collimated by a second 10X objective lens (element 10 of Supporting Fig. 1) with NA=0.25 and WD=7mm. Then, the laser light is reflected on a dichroic mirror (element 11) and guided to the four quadrant photo-detector (element 12), placed at the end of the optical path. At the same time, the second objective focuses the white light (element 13) traveling in the opposite direction over the slide, playing the role of a condenser in the imaging system of our setup simultaneously. Finally, the image of the trapping plane will be formed on the screen of a CCD camera (element 14).

Supporting Note 3. Trap stiffness measurements through Power Spectrum Density (PSD) method

For the experimental calibration of trap stiffness constant $\kappa_{x/y}$, we employ the Power Spectrum Density (PSD) method,[15,16] using the four-quadrant photo-detector (element 12 of Supporting Fig. 1). This process, stores, during a measurement time of one second, 65536 values of voltage differences between the 4 photo-detectors in x and y, which are calculated as S_x and S_y of Supporting Fig. 4.

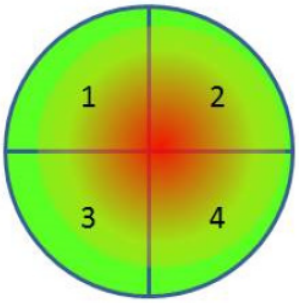

$$S_x = (V_2 + V_4) - (V_1 + V_3)$$
$$S_y = (V_1 + V_2) - (V_3 + V_4)$$

Supporting Figure 4: **Sketch of a four-quadrant photo-detector.** Representation of the four-quadrant photo-detector detecting the intensity of a Gaussian incident light beam. The labeling of each quadrant guides the sub-indices of the voltages of S_x and S_y.

Then if we Fourier transform this data we have a power spectrum of the oscillation frequencies of the particles inside the trap. The theoretical dynamics of a particle in a harmonic potential undergoing Brownian motion states that its power spectrum has a Lorentzian function shape. Therefore, by fitting the experimental power spectrum as a Lorentzian function, we can describe the properties of our harmonic potential, including the κ parameter. After the Lorentzian fitting of the particle oscillation data, we will be able to extract the corner frequency f_c, which is the frequency at which the power of the samples starts to decay. Identifying this frequency, we can use the relation $\kappa_i = 2\,\pi\,\beta\,f_c$, where $\beta = 6\pi\nu a$ is the friction coefficient, ν is the viscosity coefficient of the medium and a the radius of the particle. In Supporting Fig. 5 we show examples of 6 κ_x measurements, where the data obtained with

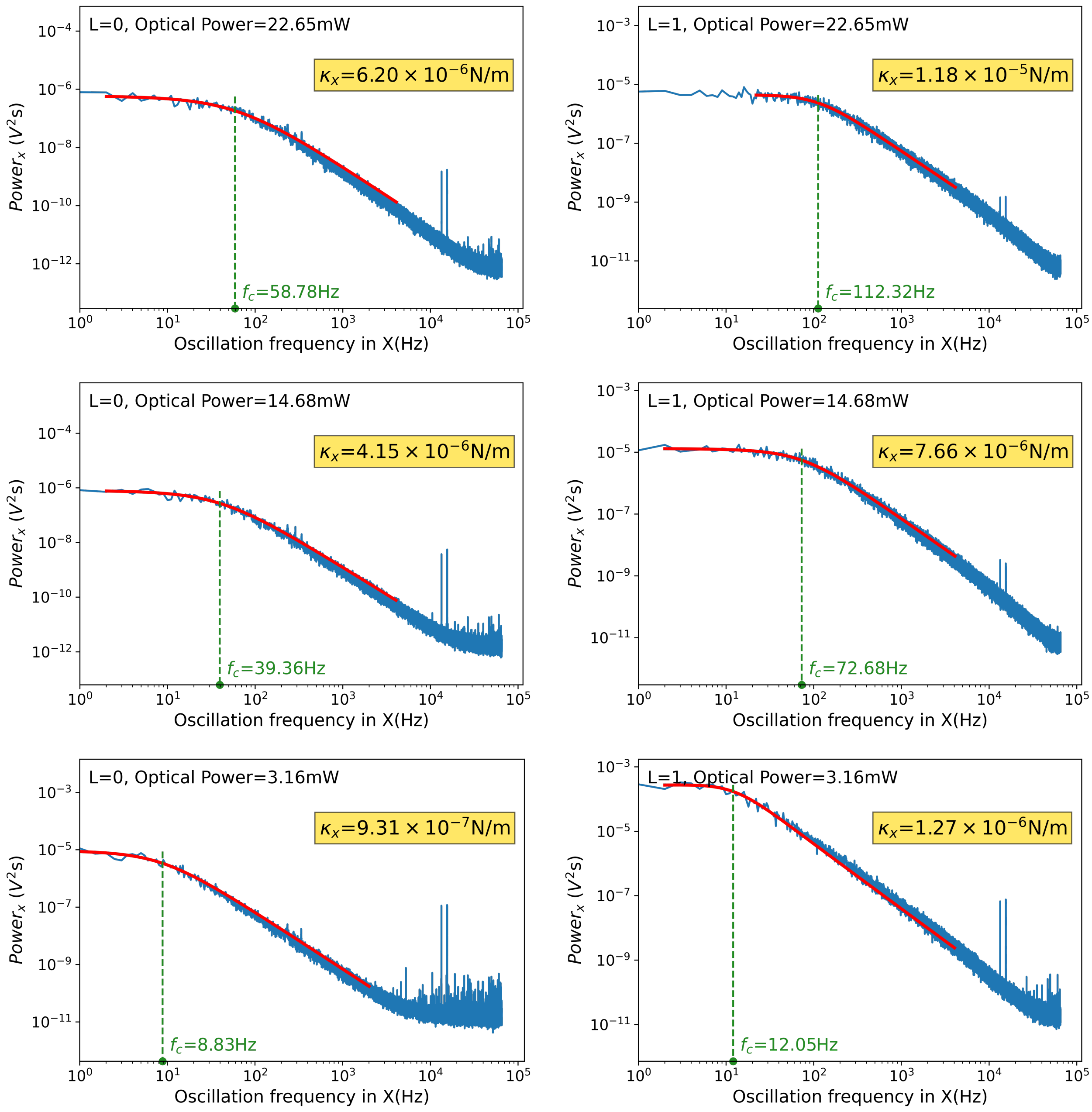

Supporting Figure 5: **Example of PSD procedure for stiffness constant calculation.** Examples of 6 different κ_x measurements: 3 optical powers with incident light beam with topological charge $L = 0$ and another 3 with $L = 1$. It can be observed the processing of the particle oscillation frequencies in order to extract the κ_x parameter. First by applying the Lorentzian fitting and then by determining the f_c.

the four-quadrant photo-detector is processed in order to obtain the stiffness parameter of the optical trap.

References

(1) Rose, M. *Multipole Fields*; Structure of matter series; Wiley, 1955; isbn: 9780598538802.

(2) Jackson, J. D. *Classical Electrodynamics: Third Edition*; American Association of Physics Teachers, 1999; isbn: 978-0-471-30932-1.

(3) Tischler, N.; Zambrana-Puyalto, X.; Molina-Terriza, G. The role of angular momentum in the construction of electromagnetic multipolar fields. *European Journal of Physics* **2012**, *33*, 1099.

(4) Novotny, L.; Hecht, B. *Principles of Nano-Optics*; Cambridge University Press, 2006; doi:10.1017/CBO9780511813535.

(5) Zambrana-Puyalto, X.; Vidal, X.; Molina-Terriza, G. Excitation of single multipolar modes with engineered cylindrically symmetric fields. *Opt. Express* **2012**, *20*, 24536–24544.

(6) Tung, W.-K. *Group Theory in Physics*; World Scientific, 1985; doi: 10.1142/1279.

(7) Zambrana-Puyalto, X.; D'Ambrosio, D.; Gagliardi, G. Excitation Mechanisms of Whispering Gallery Modes with Direct Light Scattering. *Laser & Photonics Reviews* **2021**, *15*, 2000528.

(8) Lorenz, L. *Lysbevægelsen i og uden for en af plane Lysbølger belyst Kugle*; na, 1890; doi: 10.1140/epjh/e2019-100021-6.

(9) Lorenz, L. Sur la lumière réfléchie et réfractée par une sphère transparente. *Oeuvres Scientifiques* **1898**, 405–529.

(10) Mie, G. Beiträge zur Optik trüber Medien, speziell kolloidaler Metallösungen. *Annalen der Physik* **1908**, *330*, 377–445.

(11) Gouesbet, G.; Gréhan, G. *Generalized lorenz-mie theories*; Springer, 2011; Vol. 31; doi: 10.1007/978-3-319-46873-0.

(12) Ye, Q.; Lin, H. On deriving the Maxwell stress tensor method for calculating the optical force and torque on an object in harmonic electromagnetic fields. *European Journal of Physics* **2017**, *38*, 045202.

(13) Neves, A. A. R.; Cesar, C. L. Analytical calculation of optical forces on spherical particles in optical tweezers: tutorial. *J. Opt. Soc. Am. B* **2019**, *36*, 1525–1537.

(14) Mahamdeh, M.; Campos, C. P.; Schäffer, E. Under-filling trapping objectives optimizes the use of the available laser power in optical tweezers. *Opt. Express* **2011**, *19*, 11759–11768.

(15) Ghislain, L. P.; Switz, N. A.; Webb, W. W. Measurement of small forces using an optical trap. *Review of Scientific Instruments* **1994**, *65*, 2762–2768.

(16) Gittes, F.; Schmidt, C. F. *Chapter 8 Signals and Noise in Micromechanical Measurements*; Academic Press, 1997; Vol. 55; doi: 10.1016/S0091-679X(08)60406-9.
